# Dietary Agaro-Oligosaccharides Are Associated with Higher Colonic Mucin-Related Marker Levels in Mice and *MUC2* mRNA Expression in HT-29 Cells

**DOI:** 10.3390/foods15213927

**Published:** 2026-11-02

**Authors:** Hideaki Takahashi, Tadashi Fujii, Koji Karasawa, Yu Koyanagi, Tomoki Tanabe, Akihito Endo, Mamoru Tanaka, Shoei Okuda, Hibiki Yamaguchi, Kotoyo Fujiki, Kohei Funasaka, Eizaburo Ohno, Yoshiki Hirooka, Chikako Yamada, Takumi Tochio

**Affiliations:** 1 Department of Medical Research on Prebiotics and Probiotics, Fujita Health University, Toyoake 470-1192, Japan; 2 BIOSIS Lab. Co., Ltd., Toyoake 470-1192, Japan; 3 Department of Gastroenterology and Hepatology, Fujita Health University, Toyoake 470-1192, Japan; 4 Research & Development Department, Ina Food Industry, Co., Ltd. Ina, Nagano 399-4498, Japan; 5 Department of Nutritional Science and Food Safety, Faculty of Applied Bioscience, Tokyo University of Agriculture, Tokyo 156-8502, Japan; 6 Graduate School of Bioscience and Biotechnology, Chubu University, 1200 Matsumoto, Kasugai 487-8501, Japan; 7 Graduate School of Nutritional Sciences, Nagoya University of Arts and Sciences, Nisshin 470-0196, Japan; 8 Department of Gastroenterology and Hepatology, Fujita Health University Okazaki Medical Center, Okazaki 444-0827, Japan

**Keywords:** agaro-oligosaccharides, mucin, *Akkermansia muciniphila*, gut microbiota, HT-29 cells

## Abstract

Agaro-oligosaccharides (AOSs) are agar-derived oligosaccharides composed of D-galactose and 3,6-anhydro-L-galactose. This study investigated the effects of dietary AOS on colonic mucin-related markers and their relationships with the cecal microbiota and short-chain fatty acids (SCFAs) in normal mice. Female BALB/c mice received a control diet or a diet with 5% cellulose replaced by AOS for 28 days (six mice in two cages per group). Colonic mucin-related markers, cecal microbiota, and SCFAs were assessed alongside in vitro experiments using *Akkermansia muciniphila* and HT-29 cells. AOS increased colonic Alcian blue–periodic acid–Schiff-positive area, fecal mucin content, and colonic *Muc2* and *Spdef* mRNA expression. *16S rRNA* sequencing revealed higher *Akkermansia* abundance, while species-specific qPCR indicated a higher *A. muciniphila*-to-total bacterial *16S rRNA* gene copy ratio. Cecal acetate, propionate, and butyrate concentrations decreased; estimated total amounts decreased for acetate and butyrate but not significantly for propionate. In vitro, AOS increased *A. muciniphila* culture OD_660_ at 0.2–0.5% but decreased it at 1.0% and increased HT-29 *MUC2* mRNA expression after 2 h. Despite limited cage-level replication and untested barrier function and microbial causality, these findings associate dietary AOS substitution with increased colonic mucin-related markers and a short-term *MUC2* mRNA response in HT-29 cells.

## 1. Introduction

Prebiotics, defined as “a substrate that is selectively utilized by host microorganisms conferring a health benefit,” have attracted growing attention as nutritional approaches to supporting intestinal barrier function [1]. The colonic mucosal barrier is dynamically influenced by diet, the gut microbiota, and microbiota-derived metabolites. In mice colonized with a defined human gut microbiota, dietary fiber deprivation increased microbial utilization of host mucus glycans and eroded the colonic mucus barrier [2]. The colonic mucus barrier is a major component of host defense, as it physically separates luminal microbes from the epithelium [3]. MUC2, the predominant gel-forming mucin in the colon, is a major structural component of this barrier. Notably, disruption of MUC2-dependent barrier function induces spontaneous colitis in *Muc2*-deficient mice and increases susceptibility to luminal injury and infection [4,5].

Agaro-oligosaccharides (AOSs), produced by the partial hydrolysis of agarose, are even-numbered agar-derived oligosaccharides composed of alternating D-galactose and 3,6-anhydro-L-galactose (AHG) residues linked by alternating β-1,4 and α-1,3 glycosidic bonds to form repeating agarobiose units [6]. In AOS, galactose is located at the non-reducing end, whereas AHG is located at the reducing end. The microbiota-related properties of AOS are distinct from those of classical prebiotics, which are generally characterized by the enrichment of beneficial bacteria such as *Bifidobacterium* and *Lactobacillus*. A previous in vitro study showed that AOS inhibited the growth of *Mediterraneibacter gnavus* (formerly *Ruminococcus gnavus*) and *Fusobacterium nucleatum* while preserving *Bifidobacterium longum*, *B. adolescentis*, *Lactiplantibacillus plantarum*, and *Lacticaseibacillus casei* [7]. In contrast, *Bacteroides uniformis* has been reported to degrade AOS [8,9], suggesting that AOS can also be utilized by specific gut bacteria. These findings suggest that, unlike classical prebiotics that mainly promote the growth of beneficial bacteria, AOS may selectively remodel the gut microbiota by suppressing certain bacterial taxa while promoting or allowing the expansion of AOS-utilizing bacteria. AOSs have also shown beneficial effects in experimental models of intestinal inflammation and injury, including 2,4,6-trinitrobenzene sulfonic acid-induced colitis, nonsteroidal anti-inflammatory drug-induced small intestinal injury, high-fat diet-associated dysbiosis, colon tumorigenesis, and deoxynivalenol-induced intestinal dysfunction [10,11,12,13,14]. In addition to selectively modulating the gut microbiota, AOS may directly modulate host cell responses. For example, AOSs have been reported to induce heme oxygenase-1 and suppress tumor necrosis factor-α production in stimulated immune cells [6,11]. AOSs have also been reported to attenuate TNF-α-induced myostatin expression and myosin heavy-chain protein loss in C2C12 myotubes [15]. Collectively, these findings suggest that AOS may exert intestinal effects through multiple mechanisms, including microbiota-associated and host-directed pathways.

Against this background, it remains unclear whether AOS-associated changes in the gut microbiota and microbiota-derived metabolites are linked to basal colonic mucin-related indices and whether AOS can directly induce mucin-related epithelial responses. Therefore, to address these questions in a preclinical in vivo setting, we used normal mice to investigate the effects of dietary AOS on colonic mucin-related indices, the cecal microbiota, and cecal short-chain fatty acids (SCFAs). We further performed complementary in vitro experiments to assess microbial and epithelial responses to AOS.

## 2. Materials and Methods

### 2.1. Animals and Ethics

Five-week-old female BALB/c mice (15–20 g) were purchased from Japan SLC Inc. (Hamamatsu, Japan) and housed three per cage, with two cages per dietary group, under controlled conditions (24 ± 2 °C; 12-h light/dark cycle; constant airflow), with ad libitum access to food and water. Female mice were selected to reduce the risk of fighting during group housing [16]. Cage positions were randomized to minimize potential environmental bias. The mice were randomly assigned to experimental groups using Microsoft Excel (version 2311; Microsoft Corporation, Redmond, WA, USA).

All animal studies were conducted in accordance with the Animal Experimentation Guidelines of Nagoya University of Arts and Sciences, and the study was reported in accordance with the ARRIVE guidelines. The study was approved by the Laboratory Animal Care Committee of Nagoya University of Arts and Sciences in accordance with the standards of the Ministry of the Environment (approval no. 153; approved on 2 October 2023).

### 2.2. Diets and Experimental Design

After 7 days of acclimatization to a semi-purified AIN-93G diet (CLEA Japan, Tokyo, Japan), the mice were randomly allocated to the control (Ctrl, *n* = 6) or AOS-fed (AOS, *n* = 6) group. The sample size was set empirically at six mice per group, consistent with the group size used for the fructan-supplemented groups in our previous study [17]. No a priori power calculation was performed. We identified the primary outcome measures before the experiment as cecal microbiota composition and fecal mucin content.

Mice in the Ctrl group were fed the AIN-93G diet, whereas those in the AOS group were fed a diet in which 5% (*w*/*w*) cellulose was replaced with 5% (*w*/*w*) AOS (Ina Food Industry Co., Ltd., Ina, Nagano, Japan). The supplier confirmed that the AOS preparation had the same formulation as that reported by Shirai et al. [15]: 41.8% agarobiose, 41.0% agarotetraose, 14.5% agarohexaose, and 2.7% agaro-octaose, corresponding to degrees of polymerization of 2, 4, 6, and 8, respectively, as determined by HPLC in that report. The corresponding molar masses, calculated from their molecular formulas, are approximately 324.3, 630.5, 936.8, and 1243.1 g/mol, respectively. The 5% inclusion level was consistent with the AOS-for-cellulose formulation used by Kuramitsu et al. in rats with the same reported oligosaccharide composition [18].

The feeding period was 28 days. Body weight was monitored throughout the feeding period. No body-weight loss or visible abnormalities were observed in any mouse during the feeding period. No animals were excluded, and all 12 mice (six per group) were included in the animal-level analyses. Food intake was recorded per cage and was therefore presented descriptively rather than subjected to statistical analysis. At necropsy, cecal content weight was measured. The mice used in the present study were identical to those reported previously [7]. Cecal content weight, mucin-related markers, and SCFAs are reported here for the first time. Blinding was not performed.

### 2.3. Sample Collection

During days 26–28 of the feeding period, the mice were placed in individual cages for 3 h (10:00–13:00 h) to facilitate fecal collection. Fecal samples were pooled across three consecutive days for each mouse and stored at −80 °C until analysis.

On day 28, the mice were anesthetized by isoflurane inhalation. Adequate anesthesia was confirmed by loss of the righting reflex and absence of withdrawal responses to toe, tail, or ear pinching. The mice were euthanized by cardiac puncture. The entire colon was excised and gently flushed with ice-cold phosphate-buffered saline (PBS). Colon tissues were processed for histological analysis and RNA extraction. Cecal contents were collected, snap-frozen in liquid nitrogen, and stored at −80 °C until analysis.

### 2.4. RNA Extraction from Mouse Tissues and RT-qPCR

RNA extraction, reverse transcription, and quantitative PCR were performed as previously described, with minor modifications [17]. Briefly, colon tissue was homogenized using a BioMasher II microtissue homogenizer (DWK Life Sciences, Millville, NJ, USA). Total RNA was extracted using an RNeasy Mini Kit (Qiagen, Hilden, Germany) and reverse-transcribed using a High-Capacity RNA-to-cDNA Kit (Thermo Fisher Scientific, Waltham, MA, USA). RT-qPCR was performed using a QuantStudio 3 Real-Time PCR System (Thermo Fisher Scientific) with PowerTrack SYBR Green Master Mix (Thermo Fisher Scientific) according to the manufacturer’s instructions. Primer sequences were as follows: *Muc2* forward 5′-TGCTGACGAGTGGTTGGTGAATG-3′ and reverse 5′-TGATGAGGTGGCAGACAGGAGAC-3′ [19]; *SAM-pointed domain-containing ETS transcription factor* (*Spdef*) forward 5′-GTTGCCTGCTACTGTTCCCAGATG-3′ and reverse 5′-AAAGCCACTTCTGCACGTTACCAG-3′ [20]; and *18S rRNA* forward 5′-TTGGAGGGCAAGTCTGGTG-3′ and reverse 5′-CCGCTCCCAAGATCCAACTA-3′ [17]. The cycling program consisted of an initial hold at 95 °C for 2 min, followed by 40 cycles at 95 °C for 15 s and 60 °C for 30 s. Melt curve analysis was performed to confirm amplification specificity. Relative gene expression was calculated using the 2^−ΔΔCt^ method and normalized to 18S rRNA [21].

### 2.5. Quantification of Fecal Mucin

Fecal mucin levels were quantified using a commercially available assay kit (Cosmo Bio Co., Ltd., Tokyo, Japan), as previously described [22]. Briefly, pooled fecal samples were processed according to the manufacturer’s instructions, and fluorescence intensity was measured at excitation and emission wavelengths of 336 and 383 nm, respectively, using a microplate reader (POWERSCAN HT; BioTek Instruments, Winooski, VT, USA). Fecal mucin content was expressed as milligrams per gram of dry feces.

### 2.6. Histological Analysis

Histological analyses were performed as previously described [22]. Briefly, colon tissues were fixed in 4% paraformaldehyde in PBS for 7 days, embedded in paraffin, sectioned at a thickness of 4 μm, and stained with Alcian blue–periodic acid–Schiff (AB-PAS) to visualize goblet cell-associated mucins. The stained sections were imaged using an inverted microscope (Nikon Eclipse Ti-U; Nikon, Tokyo, Japan) equipped with NIS-Elements software (v4.30). For quantitative analysis, the AB-PAS-positive area was expressed as a percentage of the total tissue area using BZ-II Analyzer software (Keyence, Osaka, Japan).

### 2.7. 16S rRNA Gene Sequencing Data and Bioinformatic Analysis

The raw *16S rRNA* gene sequencing dataset generated from cecal DNA was identical to that used in our previous study [7] and is available in the NCBI Sequence Read Archive under BioProject accession PRJNA1130056. The previous study reported the Chao1 and Shannon indices, principal coordinate analysis (PCoA) based on generalized Jensen–Shannon distances, family-, genus-, and species-level taxonomic profiles, family-level LEfSe, and genus-level LEfSe restricted to the family *Lachnospiraceae*. In the present study, the same raw dataset was reanalyzed using a different analytical pipeline to assess Observed Features, weighted UniFrac-based PCoA, genus-wide LEfSe to identify genera that discriminated between the Ctrl and AOS groups, and correlations between discriminative genera and the newly assessed mucin-related markers. In addition, the relative abundance of *A. muciniphila* was assessed by species-specific qPCR.

Bioinformatic analyses were performed using QIIME 2 version 2023.5 [23]. Sequence quality control and denoising were performed using DADA2 [24], with truncation parameters set to --p-trunc-len-f 240 and --p-trunc-len-r 240. Taxonomic assignment was performed using the q2-feature-classifier classify-sklearn method [25], with Greengenes2 version 2022.10 as the reference database [26]. Alpha- and beta-diversity metrics were calculated using the q2-diversity core-metrics-phylogenetic pipeline. PCoA was performed based on weighted UniFrac distances. Group differences in beta diversity were assessed using permutational multivariate analysis of variance (PERMANOVA) with q2-diversity beta-group-significance. Differentially abundant taxa between the Ctrl and AOS groups were identified using linear discriminant analysis effect size (LEfSe) [27]. Taxa with an LDA score > 4.0 were considered discriminative features.

### 2.8. Quantitative Analysis of Cecal A. muciniphila by qPCR

Quantitative PCR (qPCR) was performed to determine *A. muciniphila*-specific and total bacterial 16S rRNA gene copy numbers. Amplification was performed using a QuantStudio 3 Real-Time PCR System (Thermo Fisher Scientific, Waltham, MA, USA) with PowerTrack SYBR Green Master Mix (Thermo Fisher Scientific) according to the manufacturer’s instructions. The qPCR protocol comprised an initial denaturation at 95 °C for 2 min, followed by 40 cycles at 95 °C for 10 s, 60 °C for 15 s, and 72 °C for 15 s and a final extension at 72 °C for 1 min. The *A. muciniphila*-specific primer pair consisted of AM1 (forward, 5′-CAGCACGTGAAGGTGGGGAC-3′) and AM2 (reverse, 5′-CCTTGCGGTTGGCTTCAGAT-3′) [28]. Total bacterial 16S rRNA gene abundance was quantified using the forward primer 5′-CGGTGAATACGTTCCCGG-3′ and reverse primer 5′-TACGGCTACCTTGTTACGACTT-3′ [29]. Absolute quantification was performed using PCR amplicons generated from *A. muciniphila* JCM 33894^T^ DNA as external standards for the *A. muciniphila* assay and PCR amplicons generated from cecal DNA as external standards for the total bacterial 16S rRNA gene assay. The relative abundance of *A. muciniphila* was calculated as the ratio of *A. muciniphila*-specific 16S rRNA gene copy number to total bacterial 16S rRNA gene copy number and multiplied by 10^4^.

### 2.9. SCFA Analysis in Cecal Contents

Briefly, SCFA concentrations, including those of acetate, propionate, and butyrate, in the cecal samples were analyzed as previously described [17]. The carboxyl groups of the SCFAs in each sample were labeled with 2-nitrophenyl hydrazide using short- and long-chain fatty acid analysis kits (Cat. No. XSSFACR01; YMC, Kyoto, Japan) according to the manufacturer’s instructions. SCFA concentrations were expressed as μmol/g wet cecal content. The estimated total amount of each SCFA per cecum was calculated as follows: estimated total SCFA amount (μmol/cecum) = SCFA concentration (μmol/g wet cecal content) × total wet weight of the cecal contents from the same mouse (g).

### 2.10. BLASTp Analysis of Proteins Related to AHG Release and Catabolism

The amino acid sequences of glycoside hydrolase family 117 (GH117; VejGH117; WP_014232194.1; 360 amino acids) [30], 3,6-anhydro-L-galactose dehydrogenase (AHGD; WP_014232205.1; 480 amino acids), and 3,6-anhydro-L-galactonate cycloisomerase (ACI; WP_014232231.1; 362 amino acids) [31,32], all from *Vibrio* sp. EJY3, were used as reference queries. *B. uniformis* NBRC 113350 was included as a genomic comparator because its plasmid pBUN1 has been reported to contain an agarose-utilization locus [33]. The other comparison strain was *A. muciniphila* JCM 33894^T^, which was used in the culture experiments.

Separate protein–protein BLAST (BLASTp) searches were performed using the NCBI web interface against the non-redundant protein sequence database (nr; accessed 21 October 2026), with organism filters restricted to *B. uniformis* (taxid: 820) or *A. muciniphila* (taxid: 239935). The blastp algorithm was used with an Expect threshold of 0.05, a maximum of 5000 target sequences, and no additional exclusion filters. Returned hits were assigned to the comparison strains using accession mappings from the corresponding fixed RefSeq genome assemblies, associated feature annotations, and Identical Protein Group records (Table S1). For each query and strain, the highest-bit-score returned RefSeq hit assigned to that strain was reported, without additional sequence-identity or query-coverage cutoffs. Query sequences, RefSeq assembly accessions, and search conditions are provided in Table S1. Table 1 summarizes query coverage and sequence identity; E-values, bit scores, protein accession numbers, and annotations are provided in Table S2.

### 2.11. In Vitro Growth Test of A. muciniphila in the Presence of AOS

*A. muciniphila* JCM 33894^T^ was obtained from the Japan Collection of Microorganisms (JCM), RIKEN, Tsukuba, Japan, and used in this study. The strain was routinely precultured in Gifu Anaerobic Medium (GAM; Shimadzu Diagnostics, Tokyo, Japan) broth at 37 °C for 16 h. The precultured cells were inoculated into GAM broth supplemented with various concentrations of AOS (0, 0.1, 0.2, 0.5, and 1.0%). The AOS concentration range was selected with reference to our previous bacterial growth-inhibition study [7] to examine the concentration-dependent growth response of *A. muciniphila*. All broths used in this study were degassed by boiling and pre-reduced by purging with N_2_ gas before autoclaving, as previously described [34]. The cultures were incubated at 37 °C for 24 h, and the optical density of the cultures was measured at 660 nm using a spectrophotometer (U-2900; Hitachi, Tokyo, Japan). For each AOS concentration, six separate cultures were prepared (*n* = 6).

### 2.12. HT-29 Cell Culture and AOS Treatment

HT-29 cells were cultured as previously described [35]. HT-29 human colon adenocarcinoma cells (KAC Co., Ltd., Kyoto, Japan) were routinely maintained in McCoy’s 5A medium supplemented with 10% fetal bovine serum under standard culture conditions in a humidified atmosphere containing 5% CO_2_ at 37 °C. For the experiment, cells from the same parent culture were seeded in 6-well plates (9.5 cm^2^/well) at 5.1 × 10^5^ cells/well in 3 mL of medium (1.7 × 10^5^ cells/mL). AOS was added to the culture medium at final concentrations of 12.5, 25, and 50 μg/mL. The tested concentration range overlapped with those used in previous AOS cell studies using RAW264 macrophages (10–200 μg/mL) [11] and Vero cells (12.5–50 μg/mL) [36]. Equal volumes of PBS were added to the control wells. Based on a previous study assessing mucin gene expression in HT-29 cells [37], the cells were incubated for 2 h before RNA extraction to assess the *MUC2* mRNA response. No other exposure durations were tested. The experiment was performed once, with three separately treated wells per condition. RNA was extracted from each well separately.

### 2.13. RNA Extraction from HT-29 Cells, cDNA Synthesis, and RT-qPCR

Total RNA was extracted from the cells using a NucleoSpin RNA Kit (Takara Bio, Kusatsu, Shiga, Japan) according to the manufacturer’s instructions. RNA quantity and purity were assessed using a NanoDrop spectrophotometer (Thermo Fisher Scientific), and RNA integrity was confirmed by agarose gel electrophoresis. Total RNA (1 μg) was reverse-transcribed using M-MLV Reverse Transcriptase (Thermo Fisher Scientific). Quantitative PCR was performed using a QuantStudio 3 Real-Time PCR System with SYBR Green qPCR Master Mix (Thermo Fisher Scientific). The *MUC2* primer set was designed using Primer-BLAST (National Center for Biotechnology Information, https://www.ncbi.nlm.nih.gov/tools/primer-blast/ (accessed on 8 April 2025)). Primer sequences for *MUC2* were as follows: forward, 5′-TGTAGGCATCGCTCTTCTCA-3′; reverse, 5′-GCCCACTCCATCTACCACAG-3′. *GAPDH* was amplified using the Human *GAPDH* Primer included in the Human Housekeeping Gene Primer Set (Takara Bio, Kusatsu, Shiga, Japan) according to the manufacturer’s instructions and was used as the reference gene. The thermal cycling conditions were as follows: 95 °C for 10 min, followed by 40 cycles at 95 °C for 15 s and 60 °C for 1 min. Melt curve analysis was performed to confirm amplification specificity. Relative gene expression was calculated using the 2^−ΔΔCt^ method and normalized to *GAPDH* expression.

### 2.14. Statistical Analysis

Statistical analyses were performed using GraphPad Prism version 11.0.2 (GraphPad Software, Boston, MA, USA). Comparisons between the two groups were performed using unpaired *t*-tests for parametric data and SCFA data. Body-weight changes were compared between groups separately on days 7, 14, 21, and 28 using unpaired *t*-tests. Microbiome-related group comparisons were performed using the Mann–Whitney U test, and PERMANOVA was used for beta-diversity comparisons. *A. muciniphila* culture data and HT-29 cell data were analyzed using one-way ANOVA followed by Dunnett’s multiple-comparison test. Correlations between mucin-related markers and other variables were evaluated using Spearman’s rank correlation. The resulting *p*-values were adjusted using the Benjamini–Hochberg false discovery rate (FDR) procedure [38]. The adjusted values are reported as q-values, with *q* < 0.05 considered statistically significant for these exploratory correlations. Animal-level analyses did not account for clustering within cages. Animal-level group-comparison *p*-values are nominal, and the correlation q-values do not account for within-cage dependence or between-group separation. Statistical significance was otherwise set at *p* < 0.05.

## 3. Results

### 3.1. General Observations During the Feeding Period

**Figure 1:**
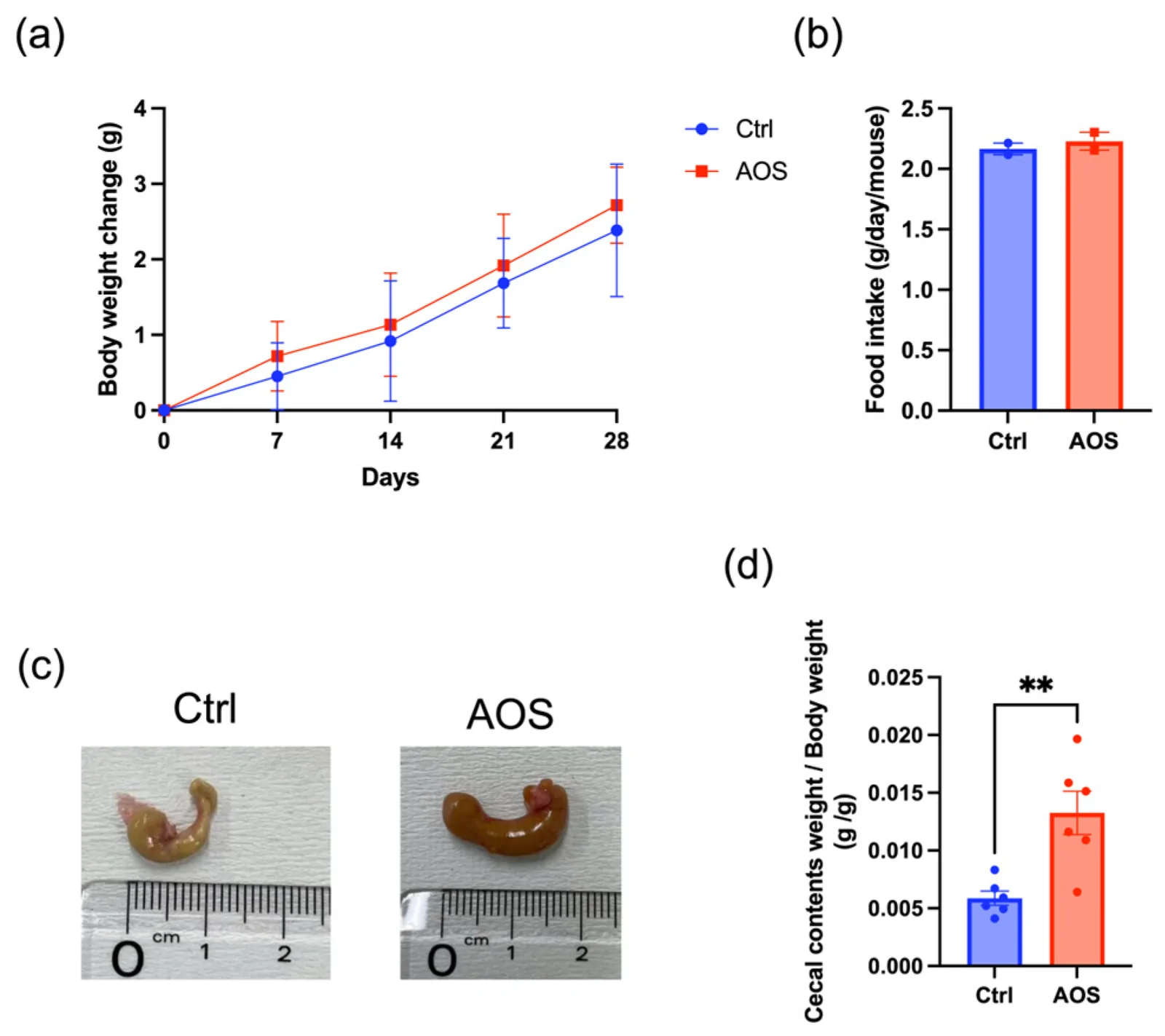
General observations in mice fed the control or agaro-oligosaccharide (AOS)-substituted diet. (**a**) Body-weight changes during the 28-day feeding period (*n* = 6 mice per group). (**b**) Mean food intake per mouse calculated from cage-level records; each point represents one cage (two cages per group). Food intake is presented descriptively. (**c**) Representative cecal images. (**d**) Cecal content weight normalized to body weight; points represent individual mice. Data are shown as mean ± standard error of the mean (SEM). The groups were compared separately on days 7, 14, 21, and 28 using unpaired *t*-tests for panel (**a**); an unpaired *t*-test was also used for panel (**d**). ** *p* < 0.01. Ctrl, control.

To characterize AOS-associated changes in colonic mucin-related markers and their relationships with the gut microbiota and SCFAs, normal mice were fed either a Ctrl or an AOS-substituted diet for 28 days. No significant between-group differences in body-weight change were detected on days 7, 14, 21, or 28 (Figure 1a). Food intake was recorded on a per-cage basis and was therefore presented descriptively without inferential statistics; no apparent between-group differences were observed (Figure 1b). Representative cecal images showed apparent cecal enlargement in the AOS group (Figure 1c). Cecal content weight normalized to body weight was significantly higher (*p* = 0.0039) in the AOS group than in the Ctrl group (Figure 1d).

### 3.2. AOS Enhances Mucin-Related Outcomes in the Colon

**Figure 2:**
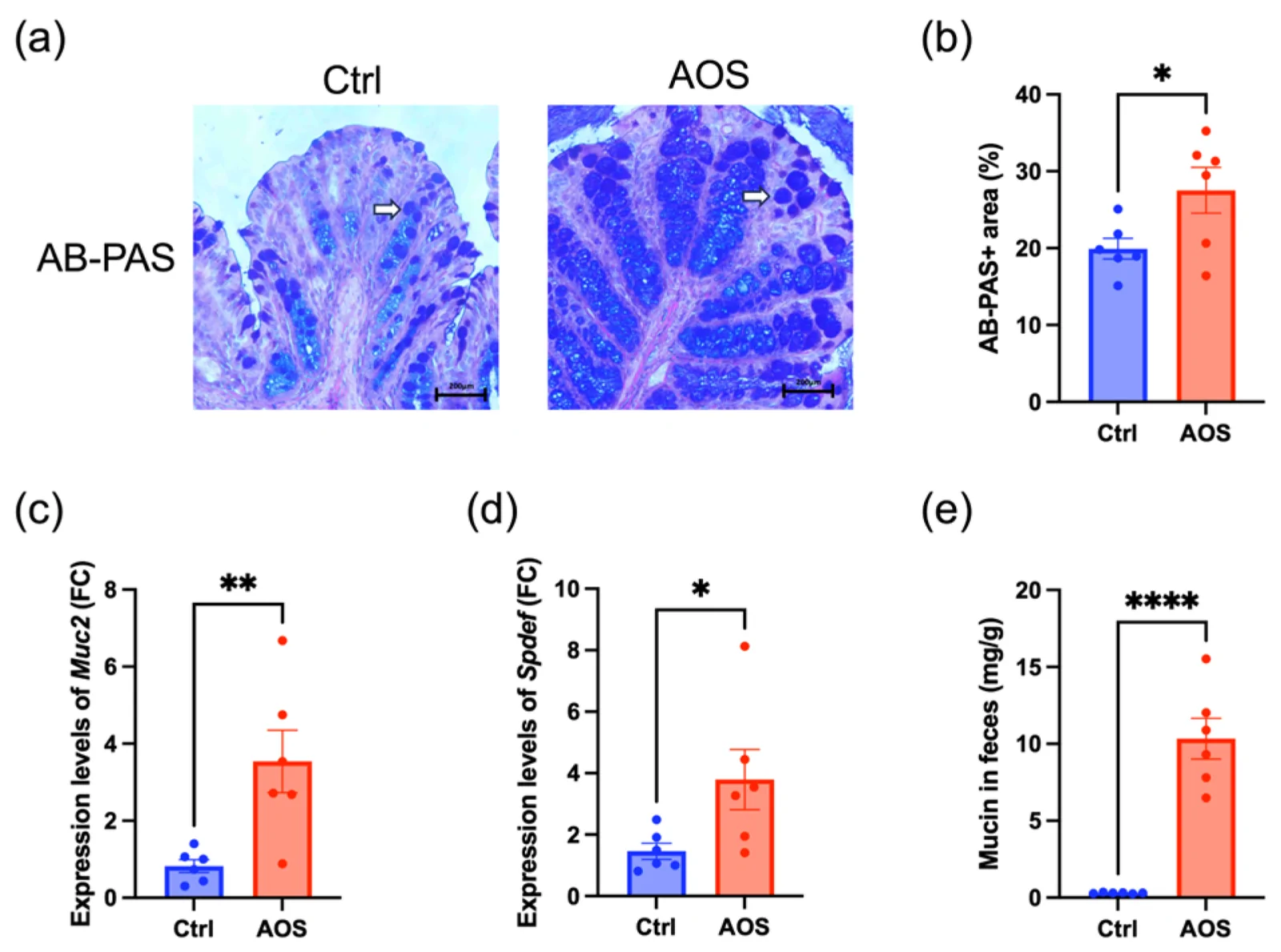
Colonic mucin-related outcomes in mice fed the control or agaro-oligosaccharide (AOS)-substituted diet. (**a**) Representative Alcian blue–periodic acid–Schiff (AB-PAS)-stained colonic sections. Arrows indicate representative AB-PAS-positive goblet cells; scale bars, 200 μm. (**b**) Quantification of the AB-PAS-positive area. (**c**) Colonic *Muc2* mRNA expression. (**d**) Colonic *Spdef* mRNA expression. (**e**) Fecal mucin content. Points represent individual mice (six mice in two cages per group), and bars represent mean ± SEM. An unpaired *t*-test was used for group comparisons. * *p* < 0.05, ** *p* < 0.01, **** *p* < 0.0001. FC, fold change; Ctrl, control.

AB-PAS staining was performed to evaluate mucin-containing goblet cells in colon tissue (Figure 2a). AB stained acidic mucins blue, whereas PAS stained neutral mucins magenta. Tissues and cells containing both neutral and acidic mucins exhibited varying shades of purple due to the combined effects of AB staining and the Schiff reagent. The AB-PAS-positive area was significantly larger (*p* = 0.0436) in the AOS group than in the Ctrl group (Figure 2b). Colonic *Muc2* mRNA expression was significantly higher (*p* = 0.0083) in the AOS group than in the Ctrl group (Figure 2c). Similarly, *Spdef* mRNA expression, a marker of goblet cell differentiation, was significantly higher (*p* = 0.0440) in the AOS group than in the Ctrl group (Figure 2d). Fecal mucin content was significantly higher (*p* < 0.0001) in the AOS group than in the Ctrl group (Figure 2e).

### 3.3. AOS Alters the Gut Microbiota and Is Associated with Akkermansia Enrichment

**Figure 3:**
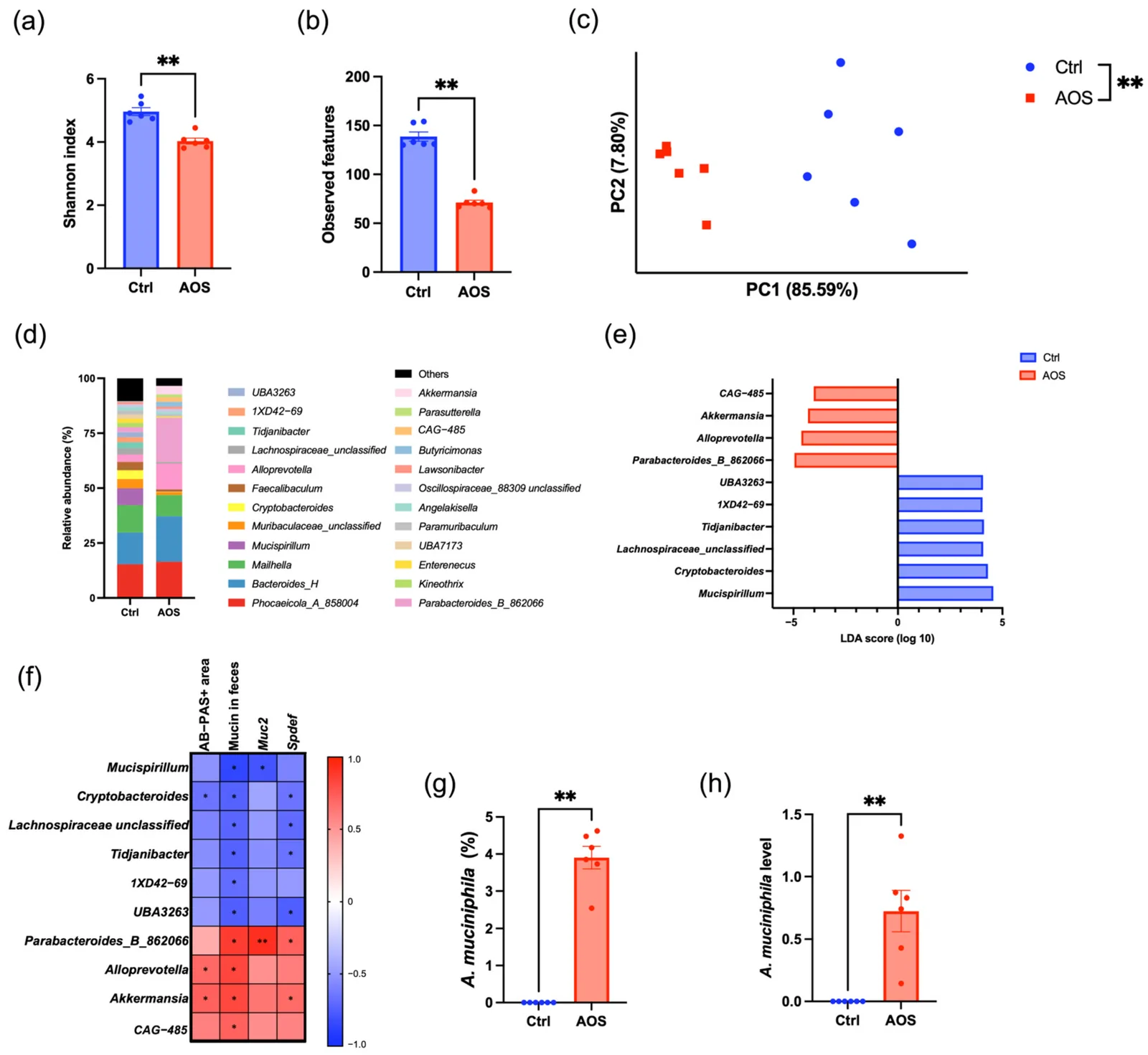
Dietary agaro-oligosaccharides (AOSs) alter the gut microbiota and are associated with *Akkermansia* enrichment. (**a**) Shannon index. (**b**) Observed Features. (**c**) Weighted UniFrac-based principal coordinate analysis. (**d**) Genus-level relative abundance; taxa with a relative abundance < 1% were grouped as “Others.” (**e**) Genus-level LEfSe analysis. (**f**) Spearman correlations between mucin-related outcomes and the 10 genus-level taxa identified by LEfSe, using pooled data from both groups. Colors indicate Spearman’s correlation coefficients. Asterisks in panel (**f**) indicate significance after Benjamini–Hochberg false discovery rate (FDR) correction across all 40 comparisons: * *q* < 0.05 and ** *q* < 0.01. (**g**) Relative abundance of *Akkermansia muciniphila_D_776786*. (**h**) *A. muciniphila* level determined by species-specific qPCR and normalized to total bacterial 16S rRNA gene abundance. Points represent individual mice. Bars in panels (**a**,**b**,**g**,**h**) show the mean ± SEM (*n* = 6 mice per group, housed in two cages per group). The Mann–Whitney U test was used for panels (**a**,**b**,**g**,**h**), and PERMANOVA was used for panel (**c**). For panels (**a**,**b**,**c**,**g**,**h**), ** *p* < 0.01. Ctrl, control.

Given the observed changes in mucin-related indices, we reanalyzed the previously reported sequencing dataset [7] to examine gut microbiota composition. Alpha diversity was significantly lower in the AOS group than in the Ctrl group, as indicated by lower Shannon index and Observed Features values (Shannon index: *p* = 0.0022; Observed Features: *p* = 0.0022; Figure 3a,b). Beta diversity analysis based on weighted UniFrac distances showed significant separation between the groups in principal coordinate analysis (PCoA) (*p* = 0.005; Figure 3c). Figure 3d shows the community composition at the genus level. LEfSe identified several taxa that discriminated between the two groups (Figure 3e). *Mucispirillum*, *Cryptobacteroides*, unclassified *Lachnospiraceae*, *Tidjanibacter*, *1XD42-69*, and *UBA3263* were enriched in the Ctrl group. In contrast, the AOS group exhibited enrichment of *Parabacteroides_B_862066*, *Alloprevotella*, *Akkermansia*, and *CAG-485*. Correlation analysis revealed significant associations between several bacterial genera and mucin-related markers (Figure 3f). Among these, *Akkermansia*, a genus that includes the mucin-degrading species *A. muciniphila* [39], was positively associated with the AB-PAS-positive area (*q* = 0.0331), fecal mucin content (*q* = 0.0178), and colonic *Spdef* mRNA expression (*q* = 0.0402). The association with colonic *Muc2* mRNA expression did not remain significant after FDR correction (*q* = 0.0557). All *Akkermansia*-assigned amplicon sequence variants detected in this dataset were classified as *Akkermansia muciniphila_D_776786*, and the relative abundance of this taxon was significantly higher (*p* = 0.0022) in the AOS group than in the Ctrl group (Figure 3g). Species-specific qPCR showed that the relative abundance of *A. muciniphila*, normalized to total bacterial 16S rRNA gene abundance, was significantly higher in the AOS group than in the Ctrl group (*p* = 0.0022; Figure 3h).

### 3.4. AOS Lowers Cecal SCFA Concentrations and the Total Amounts of Acetate and Butyrate

**Figure 4:**
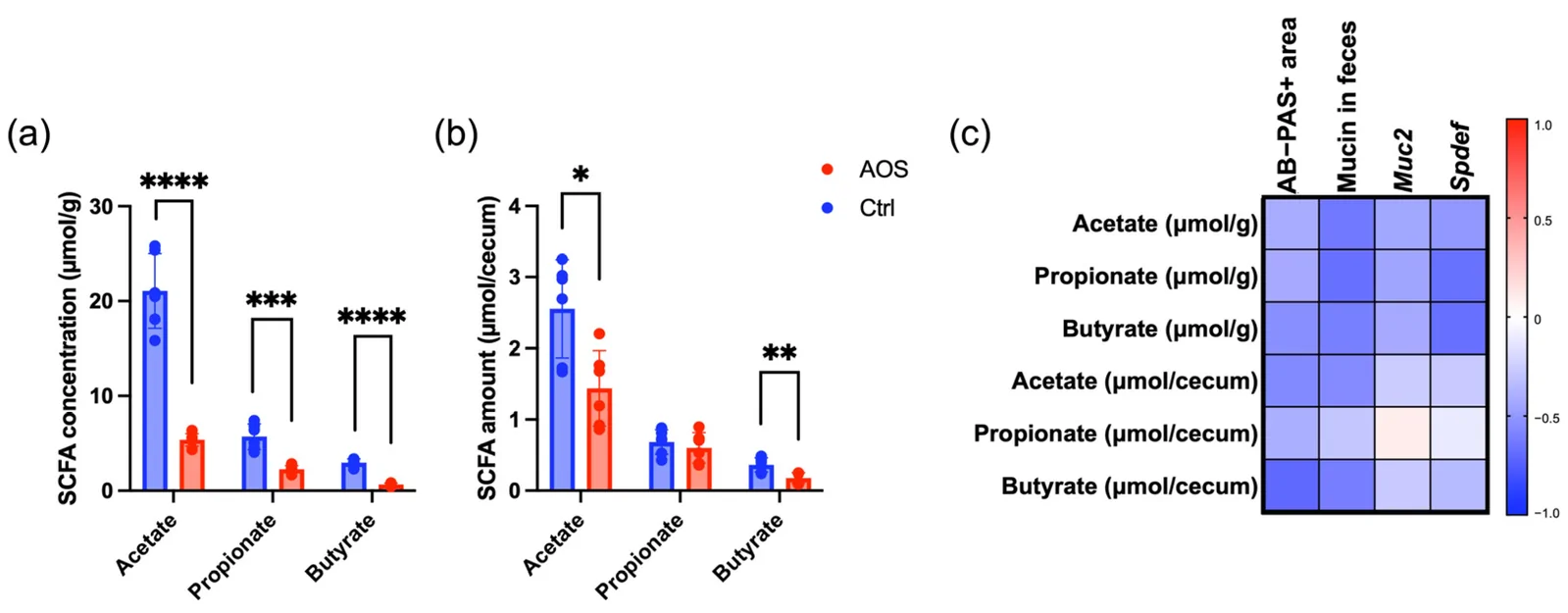
Cecal short-chain fatty acid (SCFA) concentrations, estimated total amounts, and correlations with mucin-related outcomes. (**a**) Cecal acetate, propionate, and butyrate concentrations (μmol/g wet cecal contents). (**b**) Estimated total amounts (μmol/cecum), calculated by multiplying each concentration by the total wet weight of the cecal contents from the same mouse. (**c**) Spearman correlation coefficients for SCFA concentrations and estimated amounts versus mucin-related outcomes, using pooled data from both groups. Colors indicate correlation direction and magnitude; panel (**c**) has no significance coding. Points represent individual mice, and bars represent mean ± SEM (six mice in two cages per group). Unpaired *t*-tests were used for panels (**a**,**b**). * *p* < 0.05, ** *p* < 0.01, *** *p* < 0.001, **** *p* < 0.0001. Ctrl, control.

We next examined the cecal SCFA profile. Cecal acetate, propionate, and butyrate concentrations were significantly lower in the AOS group than in the Ctrl group (acetate, *p* < 0.0001; propionate, *p* = 0.0001; butyrate, *p* < 0.0001; Figure 4a). The estimated total amounts of acetate and butyrate per cecum were also significantly lower in the AOS group (acetate, *p* = 0.0104; butyrate, *p* = 0.0048), whereas the difference in the estimated total amount of propionate was not significant (*p* = 0.4880; Figure 4b). Correlations of SCFA concentrations and estimated total amounts with mucin-related outcomes were predominantly negative and showed no consistent positive pattern (Figure 4c).

### 3.5. AOS Modulates A. muciniphila Growth In Vitro

**Figure 5:**
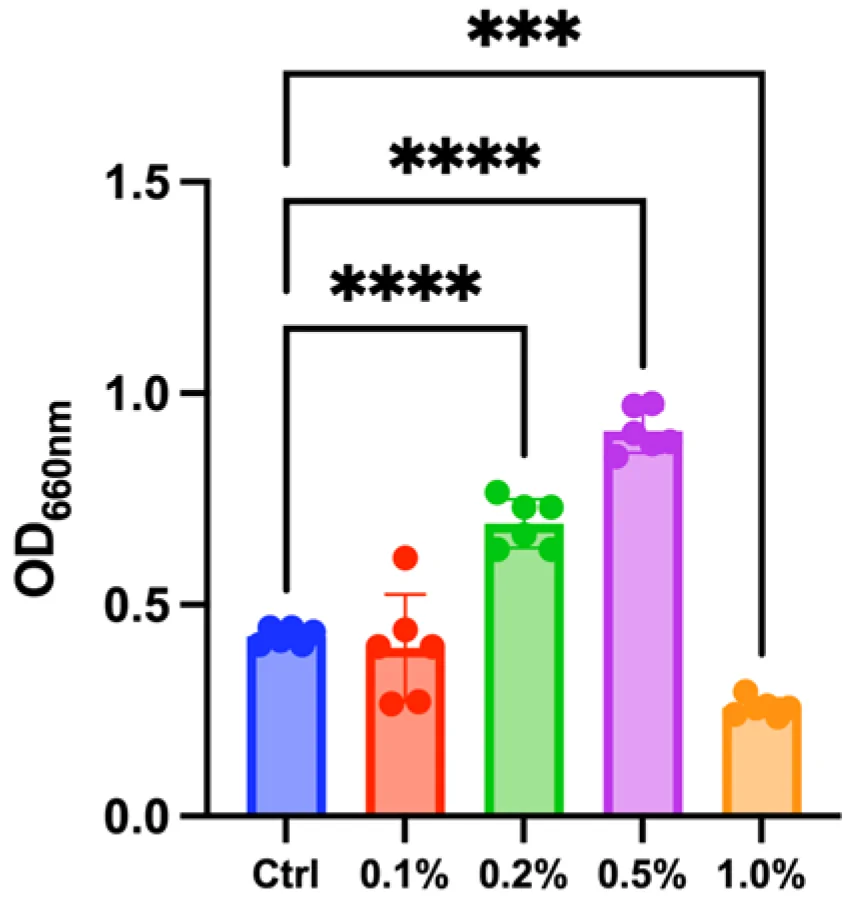
Agaro-oligosaccharides (AOSs) differentially affect *A. muciniphila* growth in vitro. Optical density at 660 nm (OD_660_) of *A. muciniphila* after 24 h of incubation in medium containing 0 (control), 0.1, 0.2, 0.5, and 1.0% AOS (*n* = 6). Points represent six separately incubated cultures per concentration, and bars represent the mean ± standard deviation (SD). Statistical analysis was performed using one-way analysis of variance (ANOVA) with Dunnett’s multiple-comparison test. *** *p* < 0.001, **** *p* < 0.0001 vs. Ctrl. Ctrl, control.

Based on the observed increase in *A. muciniphila* abundance in vivo, we examined whether AOS directly affected *A. muciniphila* growth in vitro. After 24 h of incubation, growth showed a concentration-dependent response to AOS. Compared with the Ctrl, growth was unaltered at 0.1%, significantly increased at 0.2% and 0.5%, and significantly decreased at 1.0% (0.1%, *p* = 0.8890; 0.2%, *p* < 0.0001; 0.5%, *p* < 0.0001; 1.0%, *p* = 0.0008) (Figure 5).

AOSs are composed of D-galactose and AHG. To explore the potential for AHG release and subsequent metabolism, we examined sequence similarity to three enzymes characterized in *Vibrio* sp. EJY3. During stepwise hydrolysis of agar-derived oligosaccharides, GH117 releases AHG from exposed non-reducing termini [30]. Free AHG is then oxidized by AHGD to 3,6-anhydro-L-galactonate (AHGA), which ACI converts to 2-keto-3-deoxygalactonate [31,32]. These three enzymes were therefore used as reference sequences to compare *B. uniformis* NBRC 113350 with *A. muciniphila* JCM 33894^T^.

In *B. uniformis* NBRC 113350, the selected RefSeq matches to GH117, AHGD, and ACI showed sequence identities of 61.5%, 65.2%, and 72.1%, respectively (Table 1). All three proteins were encoded on plasmid pBUN1 (NZ_AP019725.1). In *A. muciniphila* JCM 33894^T^, no significant similarity was reported for GH117 or ACI. The AHGD query yielded a significant match to Amuc_2066, with 28.2% sequence identity and 85% query coverage (Table 1). Its protein accession number, E-value, and annotation are provided in Table S2.

### 3.6. AOS Increases MUC2 mRNA Expression in HT-29 Cells

**Figure 6:**
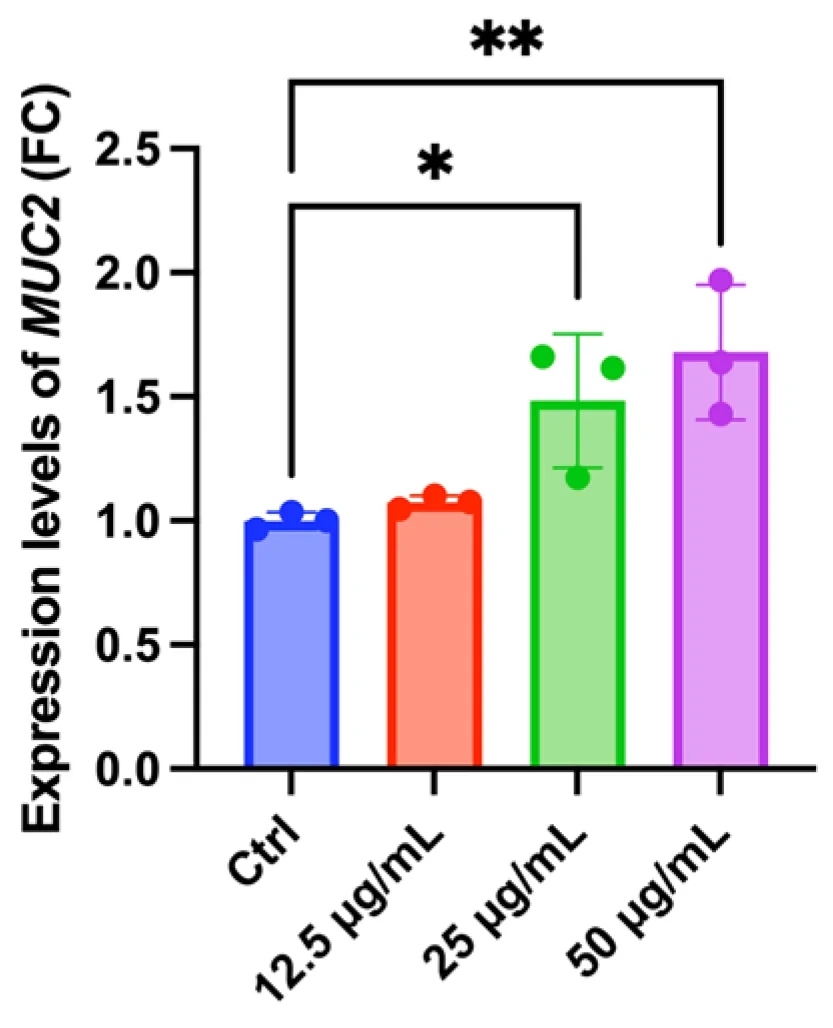
Agaro-oligosaccharides (AOSs) increase *MUC2* mRNA expression in HT-29 cells. *MUC2* mRNA expression was measured using RT-qPCR after 2 h of treatment. Points represent separately treated wells, with RNA extracted from each well separately. The experiment was performed once using cells from the same parent culture (three wells per condition, *n* = 3). Bars represent mean ± SD. Statistical analysis was performed using one-way ANOVA with Dunnett’s multiple-comparison test. * *p* < 0.05, ** *p* < 0.01 vs. Ctrl. FC, fold change; Ctrl, control.

To explore the potential direct effects of AOS on epithelial cells, we examined *MUC2* mRNA expression in HT-29 cells following AOS treatment. After 2 h, *MUC2* mRNA expression was significantly higher at AOS concentrations of 25 and 50 μg/mL, whereas no significant difference was detected at 12.5 μg/mL (12.5 μg/mL, *p* = 0.9312; 25 μg/mL, *p* = 0.0373; 50 μg/mL, *p* = 0.0065; Figure 6).

## 4. Discussion

The relationships among dietary AOS-associated changes in the gut microbiota, cecal SCFAs, and basal colonic mucin-related indices remain unclear, particularly under non-inflammatory conditions. To address this issue, we conducted in vivo studies to examine the effects of dietary AOS on colonic mucin-related outcomes, the cecal microbiota, and cecal SCFAs and complemented these analyses with in vitro assessments of microbial and epithelial responses to AOS.

No significant between-group differences in body-weight change were detected at the measured time points, and food intake showed no apparent between-group difference. In contrast, the AOS-substituted diet was associated with cecal enlargement and increased cecal content weight. The AOS group showed significant increases in the AB-PAS-positive area, fecal mucin content, and colonic *Muc2* and *Spdef* mRNA expression. To the best of our knowledge, this is the first study to report an association between AOS and colonic *Spdef* expression. SPDEF is a key transcription factor involved in goblet cell maturation and secretory lineage differentiation and positively regulates *Muc2* expression [40,41]. Therefore, the increased *Spdef* expression, AB-PAS-positive area, and *Muc2* expression raise the possibility that AOS influences goblet cell differentiation or maturation rather than only stimulating mucin release from pre-existing cells. The concurrent increases in tissue staining, fecal mucin content, and mucin-related transcripts suggest that AOS may influence both mucin synthesis and turnover.

*A. muciniphila* is a well-known mucin-degrading bacterium and an abundant resident of the human intestine [39]. In this study, *Akkermansia* was enriched in the AOS group and positively associated with the AB-PAS-positive area, fecal mucin content, and colonic *Spdef* expression after FDR correction. Species-specific qPCR also showed a higher relative abundance of *A. muciniphila* in the AOS group. Previous studies have shown that *A. muciniphila* restores mucus-layer thickness in high-fat-diet-fed mice [42] and accelerates epithelial development in intestinal stem cell-based systems [43]. *A. muciniphila* can influence host physiology through the outer membrane protein Amuc_1100, which signals through Toll-like receptor 2 (TLR2) [44,45,46]. A recent study suggested that microbiota-driven goblet cell responses may be mediated through the TLR2–*Spdef* axis [47]. These findings are consistent with the potential contribution of *Akkermansia*-derived factors, such as Amuc_1100, to increased *Spdef* and *Muc2* expression. However, this hypothesis was not validated in the present study.

In vitro experiments were performed to explore how AOS might favor *A. muciniphila* growth. AOS increased growth at intermediate concentrations but decreased it at the highest concentration tested (1.0%), suggesting that a suitable concentration range of AOS may support *A. muciniphila* growth, whereas higher concentrations may be inhibitory. The sequence comparison identified matches to all three reference enzymes in *B. uniformis* NBRC 113350. In *A. muciniphila* JCM 33894^T^, Amuc_2066 showed significant sequence similarity to the AHGD query, but its activity toward AHG remains unknown. This protein was annotated as a proline/glutamate-semialdehyde dehydrogenase, whereas no significant matches to GH117 or ACI were reported under the search conditions used (Table 1; Table S2). These results do not establish a complete AHGD–ACI pathway [31,32] in this strain. Recent enzymatic profiling of *A. muciniphila* showed that Amuc_0290, a glycoside hydrolase family 2 β-galactosidase, is a broad-acting enzyme and that Amuc_0480 and Amuc_1463, both glycoside hydrolase family 110 enzymes, display activity toward α1,3-linked galactose-containing host glycans [48]. Given the terminal β-linked D-galactose in AOS, the broad substrate specificity of Amuc_0290 makes it a candidate for testing partial AOS hydrolysis, although its activity against the AHG-containing structure of AOS remains to be established. If galactose is released, it could contribute to *A. muciniphila* growth through the annotated galactose metabolism pathway (KEGG pathway: amu00052). Previous research has shown that AHG inhibits *Streptococcus mutans* growth [49]. If partial AOS degradation releases AHG that is not further metabolized, its accumulation at higher AOS concentrations could therefore contribute to growth suppression. However, AHG was not quantified, and its growth-inhibitory effect on *A. muciniphila* has not been tested. Another possibility is a direct inhibitory effect of AOS itself, as reported for other intestinal bacteria [7]. Because this assay used GAM broth, it does not establish the direct utilization of AOS as a carbon source. Further studies measuring AOS disappearance and its breakdown products would help distinguish among these possible mechanisms.

To explore additional factors underlying the mucin-related indices, we measured SCFAs in cecal contents, as SCFAs have been implicated in mucin production [50]. Cecal acetate, propionate, and butyrate concentrations were lower in the AOS group than in the Ctrl group. The estimated total amounts were also lower for acetate and butyrate, whereas the difference in the estimated total amount of propionate was not significant. For acetate and butyrate, the lower concentrations therefore cannot be explained solely by dilution associated with increased cecal content weight. Although this finding contrasts with the classical expectation that prebiotic fibers increase luminal SCFA levels [51], it is consistent with previous AOS studies reporting altered or reduced SCFA levels despite beneficial host phenotypes [12,18]. The predominantly negative correlations with mucin-related markers further argue against an explanation based solely on increased cecal SCFA levels. However, endpoint measurements of SCFA concentrations and estimated total amounts cannot distinguish among changes in microbial production, host absorption, and luminal turnover [52].

Given that increased cecal SCFA levels did not explain the mucin-related indices and considering previous reports that AOS can directly modulate host cell responses [6,11], we performed in vitro experiments using HT-29 cells. AOS increased *MUC2* mRNA expression in HT-29 cells, supporting the possibility of a direct epithelial response to AOS. Taken together, two non-mutually exclusive pathways may explain the in vivo findings. First, AOS-associated enrichment of *A. muciniphila* may have contributed to the increases in mucin-related markers through *Akkermansia*-derived host signaling. Alternatively, AOS may have directly increased epithelial *MUC2* mRNA expression, thereby altering the mucin environment and secondarily favoring the expansion of the mucin-utilizing bacterium *A. muciniphila*. These pathways may have operated concurrently. However, the present study cannot determine their causal direction or relative contributions.

Despite these findings, several limitations should be considered. First, the dietary intervention replaced cellulose with AOS, so AOS-specific effects cannot be distinguished from those of cellulose replacement. Each dietary group comprised only two cages, and the animal-level analyses did not account for within-cage correlation, which limits the precision of the estimated dietary effects. Independent studies with adequate cage-level replication and analyses that account for cage effects are needed to confirm these findings. Only female mice and one dietary AOS level were studied; sex-dependent responses and an in vivo dose–response relationship were not evaluated. The pooled correlations may reflect differences between dietary groups rather than within-group relationships. Mechanistic experiments such as microbiota depletion, fecal transfer, TLR2 blockade, and Amuc_1100 intervention could help establish the direction of the microbiota–mucin relationship. Second, the metabolite analyses presented here focused on acetate, propionate, and butyrate. Broader targeted analyses or untargeted metabolomics, including analyses of bile acids, indole derivatives, and succinate, could identify other pathways relevant to mucin regulation. Third, increased *Muc2* mRNA expression does not necessarily indicate increased MUC2 protein abundance, and the AB-PAS-positive area does not directly measure mucus-layer thickness. MUC2 protein abundance, mucin secretion, and barrier function require direct evaluation. Finally, HT-29 is an adenocarcinoma-derived cell line, and the experiment comprised three separately treated wells from the same parent culture in a single run at one 2-h time point. Independent experiments, longer exposure times, and protein- and secretion-level measurements in more physiologically relevant epithelial models are needed. Dietary percentages cannot be directly equated with cell-culture concentrations; measurements of intestinal AOS exposure and dose–response studies would help define physiologically relevant conditions.

In conclusion, dietary AOS substitution was associated with higher levels of colonic mucin-related markers in non-inflamed mice, and AOS increased *MUC2* mRNA expression in HT-29 cells. These findings were accompanied by a higher relative abundance of *A. muciniphila* and lower cecal SCFA concentrations, with lower estimated total amounts of acetate and butyrate. Microbiota-associated and epithelial responses may both be involved, although their causal direction and relative contributions remain unresolved. Together, these results support further investigation of AOS as a dietary modulator of colonic mucin-related responses.

## Figures and Tables

**Table 1:** Sequence similarity to reference enzymes involved in AHG release and catabolism in *Bacteroides uniformis* NBRC 113350 and *Akkermansia muciniphila* JCM 33894^T^.

Query Enzyme	Query Coverage (%)	Identity (%)
*B. uniformis* NBRC 113350		
GH117	94.2	61.5
AHGD	100	65.2
ACI	99.7	72.1
*A. muciniphila* JCM 33894^T^		
GH117	—	—
AHGD	85	28.2
ACI	—	—

All queries were *Vibrio* sp. EJY3 proteins (Table S1). Values indicate query coverage and sequence identity for the highest-bit-score returned RefSeq hit assigned to each comparison strain; no additional identity or query-coverage cutoff was applied. —, no significant similarity reported under the search conditions used (Table S1). For *A. muciniphila* JCM 33894^T^, strain assignment used the reference genome registered under the equivalent designation ATCC BAA-835^T^ (GCF_000020225.1). Values are rounded to one decimal place where needed. E-values, bit scores, protein accession numbers, annotations, and calculation details are provided in Table S2. GH117, glycoside hydrolase family 117; AHG, 3,6-anhydro-L-galactose; AHGD, 3,6-anhydro-L-galactose dehydrogenase; AHGA, 3,6-anhydro-L-galactonate; ACI, 3,6-anhydro-L-galactonate cycloisomerase.

## Data Availability

The 16S rRNA gene sequencing dataset is available in the NCBI Sequence Read Archive under BioProject accession PRJNA1130056. Other data supporting the findings of this study are available from the corresponding author upon reasonable request.

## Supplementary Materials

The following supporting information can be downloaded at https://www.mdpi.com/article/10.3390/foods15213927/s1: Table S1: Reference query sequences, RefSeq assembly accessions, and Web BLASTp search conditions. Table S2: Detailed Web BLASTp results assigned to *B. uniformis* NBRC 113350 and *A. muciniphila* JCM 33894^T^.

## Abbreviations

The following abbreviations are used in this manuscript:

18S rRNA	18S ribosomal RNA
AB–PAS	Alcian blue–periodic acid–Schiff
ACI	3,6-anhydro-L-galactonate cycloisomerase
AHG	3,6-anhydro-L-galactose
AHGA	3,6-anhydro-L-galactonate
AHGD	3,6-anhydro-L-galactose dehydrogenase
ANOVA	analysis of variance
AOS	agaro-oligosaccharide
ASV	amplicon sequence variant
BLASTp	Basic Local Alignment Search Tool for proteins
Ctrl	control
DADA2	Divisive Amplicon Denoising Algorithm 2
FBS	fetal bovine serum
FC	fold change
FDR	false discovery rate
GAM	Gifu anaerobic medium
GAPDH	glyceraldehyde-3-phosphate dehydrogenase
GH117	glycoside hydrolase family 117
HO-1	heme oxygenase-1
JCM	Japan Collection of Microorganisms
LDA	linear discriminant analysis
LEfSe	linear discriminant analysis effect size
MUC2	mucin 2
NCBI	National Center for Biotechnology Information
OD_660_	optical density at 660 nm
PBS	phosphate-buffered saline
PCoA	principal coordinate analysis
PERMANOVA	permutational multivariate analysis of variance
QIIME 2	Quantitative Insights Into Microbial Ecology 2
RT-qPCR	Reverse transcription quantitative polymerase chain reaction
SCFAs	short-chain fatty acids
SD	standard deviation
SEM	standard error of the mean
SPDEF	SAM-pointed domain-containing ETS transcription factor
SRA	Sequence Read Archive
TLR2	Toll-like receptor 2
TNF-α	tumor necrosis factor-α
