# Supplementary material for: Dietary Agaro-Oligosaccharides Are Associated with Higher Colonic Mucin-Related Marker Levels in Mice and MUC2 mRNA Expression in HT-29 Cells
Source: Foods. 2026 Nov 2;15(21):3927. doi: 10.3390/foods15213927 (PMC13762985; doi:10.3390/foods15213927)
Supplement: Supplementary file 1 [file foods-15-03927-s001.zip › foods-4594820-supplementary.pdf]

Table S1. Reference query sequences, RefSeq assembly accessions, and Web BLASTp search conditions.

A. Reference query sequences

Query	Reported reaction of the reference enzyme	Source organism	Accession; length
GH117	Neoagarobiose → D-galactose + AHG	*Vibrio* sp. EJY3	WP_014232194.1<br>360 amino acids
AHGD	AHG → AHGA	*Vibrio* sp. EJY3	WP_014232205.1<br>480 amino acids
ACI	AHGA → 2-keto-3-deoxygalactonate	*Vibrio* sp. EJY3	WP_014232231.1<br>362 amino acids

B. RefSeq resources used for strain assignment

Comparison strain	RefSeq assembly accession used for strain assignment
B. uniformis NBRC 113350	GCF_006742345.1
A. muciniphila JCM 33894^T	GCF_000020225.1

The queries were the three full-length *Vibrio* sp. EJY3 reference sequences shown above. GH117-mediated AHG release was characterized by Yu et al. [30], and AHGD/ACI by the studies cited as [31,32] in the main manuscript. The reaction column describes the reference enzyme, not an experimentally established activity of a matched protein.

Web BLASTp searches were performed against nr on 21 October 2026 with organism filters restricted to *B. uniformis* (taxid: 820) or *A. muciniphila* (taxid: 239935), an Expect threshold of 0.05, a maximum of 5,000 target sequences, and no additional exclusion filters. Other settings were left at their Web defaults; individual values for the matrix, gap costs, composition-based statistics, and low-complexity filtering were not retained in the supplied output.

The listed RefSeq assemblies and associated protein records, feature annotations, and Identical Protein Group records were used to assign returned accessions to strains after the taxon-filtered nr search; they were not local BLAST databases. The *A. muciniphila* assembly is registered as ATCC BAA-835^T, an equivalent designation of JCM 33894^T, the strain used in culture.

The highest-bit-score returned RefSeq hit assigned to each comparison strain was retained without additional identity or query-coverage cutoffs. *B. uniformis* NBRC 113350 was included as a genomic comparator because pBUN1 has been reported to contain an agarose-utilization locus [33]. Detailed results are provided in Table S2. Reference numbers correspond to the main manuscript.

AHG, 3,6-anhydro-L-galactose; AHGA, 3,6-anhydro-L-galactonate; GH117, glycoside hydrolase family 117; AHGD, 3,6-anhydro-L-galactose dehydrogenase; ACI, 3,6-anhydro-L-galactonate cycloisomerase.

Table S2. Detailed Web BLASTp results assigned to *B. uniformis* NBRC 113350 and *A. muciniphila* JCM 33894^{T}.

A. Sequence-comparison results

B. uniformis NBRC 113350

Query	Selected RefSeq accession	Query coverage (%)	Identity (%)	E-value	Bit score
GH117	WP_141982224.1	94.17[a]	61.471	3×10^{-150}	431
AHGD	WP_141982230.1	100	65.21	0.0	635
ACI	WP_141982232.1	99.72[a]	72.099	0.0	534

A. muciniphila JCM 33894^{T}

Query	Selected RefSeq accession	Query coverage (%)	Identity (%)	E-value	Bit score
GH117	NSM	—	—	—	—
AHGD	WP_012421090.1	85	28.16	9×10^{-37}	146
ACI	NSM	—	—	—	—

The highest-bit-score returned RefSeq hit assigned to each strain is shown, irrespective of sequence identity and query coverage. NSM, no significant similarity reported in the saved Web BLASTp result at the reporting Expect threshold of 0.05; no numerical best E-value was supplied for these searches.

[a] Query coverage was calculated from the union of exported query-coordinate intervals divided by query length: GH117, 339/360 × 100 = 94.17%; ACI, 361/362 × 100 = 99.72%. Unmarked coverage values retain the integer percentages in the Web Descriptions export. Identity, E-value, and bit score are from the saved Web exports; identity and bit score describe the highest-bit-score alignment.

The GH117 E-value is presented as 3×10^{-150}, rounded from 2.92×10^{-150} in the saved export. E-values of 0.0 are retained as reported. All values refer to searches with the EJY3 queries listed in Table S1.

Amuc_2066 showed significant sequence similarity to the AHGD query (28.16% identity; E-value = 9×10^{-37}), but its activity toward AHG remains unknown. The matched-protein annotation is provided in part C.

B. Taxon-filtered search output counts

Organism filter	GH117	AHGD	ACI
B. uniformis	111	5	5
A. muciniphila	0	117	0

Counts are the returned entries reported in the supplied search exports, before assignment to the fixed strains. They are not counts of genes in the comparison strain or of strains in an Identical Protein Group.

Table S2. Continued.

C. Matched-protein annotations and genomic locations

Selected RefSeq accession / query	Locus tag (previous locus tag)	Replicon	Annotation of matched protein
WP_141982224.1<br>GH117	FNR01_RS20360<br>(Bun01g_38340)	plasmid pBUN1<br>(NZ_AP019725.1)	glycoside hydrolase family 117 protein
WP_141982230.1<br>AHGD	FNR01_RS20380<br>(Bun01g_38380)	plasmid pBUN1<br>(NZ_AP019725.1)	aldehyde dehydrogenase
WP_141982232.1<br>ACI	FNR01_RS20385<br>(Bun01g_38390)	plasmid pBUN1<br>(NZ_AP019725.1)	mandelate racemase/muconate lactonizing enzyme family protein
WP_012421090.1<br>AHGD	AMUC_RS11035<br>(Amuc_2066)	chromosome	bifunctional proline dehydrogenase/L-glutamate gamma-semialdehyde dehydrogenase

All three *B. uniformis* NBRC 113350 matches map to plasmid pBUN1 (NZ_AP019725.1) in GCF_006742345.1. The *A. muciniphila* match maps to the chromosome in GCF_000020225.1, registered as ATCC BAA-835^{T} (= JCM 33894^{T}).

Annotations reproduce those in the supplied sequence records and feature annotations; they do not assign the catalytic activity of the query enzyme to a matched protein. WP_012421090.1 (Amuc_2066) is annotated as a bifunctional proline dehydrogenase/L-glutamate gamma-semialdehyde dehydrogenase, rather than an experimentally verified AHG dehydrogenase.

Accession-to-strain assignment is described in Table S1. A WP accession can represent an identical sequence shared across multiple strains; the fixed assembly and locus information above specify the strain used for the present comparison.
